# Oral Lesions in the Bit Area in Finnish Trotters After a Race: Lesion Evaluation, Scoring, and Occurrence

**DOI:** 10.3389/fvets.2019.00206

**Published:** 2019-07-12

**Authors:** Kati Tuomola, Nina Mäki-Kihniä, Minna Kujala-Wirth, Anna Mykkänen, Anna Valros

**Affiliations:** ^1^Department of Production Animal Medicine, Research Centre for Animal Welfare, University of Helsinki, Helsinki, Finland; ^2^Independent Researcher, Pori, Finland; ^3^Department of Production Animal Medicine, Faculty of Veterinary Medicine, University of Helsinki, Helsinki, Finland; ^4^Department of Equine and Small Animal Medicine, Faculty of Veterinary Medicine, University of Helsinki, Helsinki, Finland

**Keywords:** animal welfare, bit, harness racing, horse, oral lesion, trotter

## Abstract

Oral lesions in the bit area are common in horses, but not comprehensively studied in harness racing horses. This study describes the type and occurrence of oral soft tissue lesions in the area affected by the bit, hereafter called the bit area, in trotters after a race. Based on our results, we suggest a system for scoring lesions according to size, type (bruise or wound), age, and depth (superficial or deep). The data was collected during a welfare program for trotters, conducted by The Finnish Trotting and Breeding Association (Suomen Hippos ry). The rostral part of the mouth of 261 horses (151 Standardbreds, 78 Finnhorses, and 32 ponies) was examined after a race in a systematic manner, using a bright light source without sedation or a mouth gag. The lip commissures (outside and inside), bars of the mandible, buccal area near the second upper premolar teeth, tongue, and hard palate were visually examined; bars of the mandible were also palpated. Points were assigned to every lesion and then added together, such that each horse got an acute lesion score. Based on the score, the horses were divided into four groups (A—D) as follows: Group A, no lesions; B, mild lesions; C, moderate lesions; D, severe lesions. Of all the horses examined, 84% (219/261) had acute lesions in the bit area. In total, 21% (55/261) had mild lesions, 43% (113/261) had moderate lesions, and 20% (51/261) had severe lesions. Visible bleeding outside the mouth was observed in 2% (6/261) of the horses. Further, 5% of the horses (13/261) had blood on the bit when it was removed from the mouth, even though no blood was visible outside the mouth. In conclusion, soft tissue lesions in the bit area were common in the Finnish trotters examined. Moreover, the absence of blood outside the mouth does not rule out serious injuries inside the mouth. The scoring system presented can be used for evaluating the severity of oral lesions in different equestrian disciplines and populations to allow for comparable data across studies.

## Introduction

Bit-related oral lesions cause pain, and are a commonly reported welfare problem for horses (1–4). Odelros and Wattle (5) reported acute soft tissue injuries in the rostral mouth in 88% (127/144) of Standardbred trotters examined in Sweden. Previous studies have also reported oral lesions in other equestrian disciplines, but dissimilar scoring systems make comparisons between studies challenging. In a study of Icelandic horses at competitions in Iceland, Björnsdottir et al. (1) reported 36% of horses with mild (up to 1 cm) lesions, and 8% with lesions that were more severe (over 1 cm) before the competition. Of these 424 horses, 77 were re-examined after the race. Bit-related lesions were found in 60%. Notably, if the horse had more than one lesion, only the most severe one was included in their data. In a study of polo and racehorses (*n* = 100) in England, Mata et al. (4) used a grading system from 0 to 5 to evaluate lip commissure and bar injuries, and another grading system to evaluate tongue injuries. In Denmark, Uldahl and Clayton (6) examined 3,143 horses from various disciplines after a competition performance (show jumping, dressage, eventing, and endurance). In total, 9.2% of the horses had lesions or visible bleeding from the mouth. However, only the corners of the mouth were examined, not the oral cavity. Lacerations of the skin and mucosa, as well as the presence of blood on the skin and mucosa were reported separately, but the four outcomes were combined into a single category for analysis.

Tell et al. (7) concluded that riding a horse with a bit and bridle can cause lesions to the oral cavity. However, they noted that oral lesions were also present in broodmares that were not regularly ridden with a bit, although to a lesser extent compared to those ridden with a bit. In this study, ulcers more than 0.5 cm in diameter were considered as large.

There have been various methods for lesion examination. After competition, Icelandic horses, polo horses, and racehorses have been examined without sedation or a mouth gag. Sedation was also not used when examining trotters in Sweden after competition, however a mouth gag and a light source was used, along with flushing the mouth with water (1, 4, 5). Tell et al. (7) performed the examination on sedated horses with a mouth gag and a light source. This examination was not related to competitions.

According to the Finnish racing guidelines, official race track veterinarians should only examine the horses after a competition if they show bleeding from the mouth. However, there is no information about the oral health of horses that do not show bleeding from the mouth after a race.

The lesion grading systems used in earlier studies are all unique to each study, and none of them account for the number, depth, and size of the lesions. We thus believe that a quick oral examination, which can be performed in the field environment and a simple, practical, and objective scoring system that includes both the number and severity of the lesions is needed to allow for more accurate comparisons among studies. Our aim was to create such a scoring system, and apply it to determine the occurrence, location, type (bruise or wound), size, depth (superficial or deep), and age (old or acute injury) of oral lesions in the bit area in a sample of trotters racing in Finland.

## Materials and Methods

### Horses

The Finnish Trotting and Breeding Association (Suomen Hippos ry) is responsible for licensing trainers, drivers, and officials, as well as creating and monitoring the racing rules. Since this study was part of the association's welfare program for trotters, the oral examination performed after the race was compulsory. The horses (*n* = 261) were privately owned trotters that participated in 10 separate harness racing events (115 races) at four race tracks in Western Finland (Pori, Tampere, Forssa, and Turku). Standardbred trotters (*n* = 151), Finnhorses (*n* = 78), and ponies (*n* = 32) ranging in age from 3 to 15 years old were included in the study. Six of the Finnhorses participated to the monté race and all other horses participated into ordinary harness races.

Initially, the horses were randomly selected from the starting lists, however practical constraints were taken into account in order to maximize the number of horses examined in the limited time after finishing the race performance. The horses evaluated in previous races were excluded so that none was evaluated more than once. The horses which were resisting the examination were excluded from the study. We later checked whether the horses competed again within 2 weeks.

### Oral Examination

The horses were examined 5–20 min after the race at their outdoor harnessing booth in the warm–up area. The rostral part of the oral cavity was evaluated by the first author of this study, who is a veterinarian experienced in oral examination of horses. The examination, which was modified from the one used in Icelandic horses (1), was carried out without sedation or a mouth gag; the horse was without the bridle, and wearing only its own halter. During the examination, the veterinarian wore disposable nitrile gloves and a Lumonite Navigator 3000 headlamp set at 420–1,300 lumens. The examination began with the examiner standing on the left side of the horse. The tongue was externally guided to the left side, allowing evaluation of the buccal mucosa near the second upper premolar tooth (106) and the mucosa at the inside of the lip commissure, on the contralateral (right) side. The tongue and palate were examined visually. If a sharp hook in 106 or 206 teeth was noticed while examining the buccal area near those teeth, it was recorded and the trainer was informed, but otherwise we could not palpate and examine sharp enamel points without mouth gag. Finally, the left bar area was palpated, and the left external commissure of the lips (the outside skin area) was examined. The same procedure was repeated on the right side of the horse (Video 1 in Supplementary Material). Video recordings of some of the typical lesions were taken with a digital camera (Panasonic DMC-GX7). An assistant recorded the findings of the oral examination and the bit type on a data sheet, which was a modified version of a former Vet Form 2 from the International Federation of Icelandic Horse Associations (Data Sheet 1 in Supplementary Material).

### Lesion Scoring

**Figure 1:**
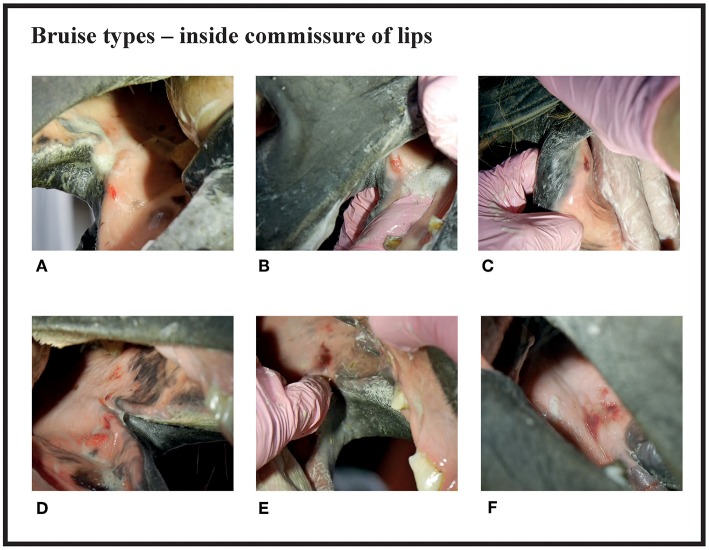
Bruise types and their points at the inside commissure of the lips. **(A)** 1 point, **(B)** 2 points, **(C)** 2 points, **(D)** 3 points, **(E)** 4 points, **(F)** 4 points.

**Figure 2:**
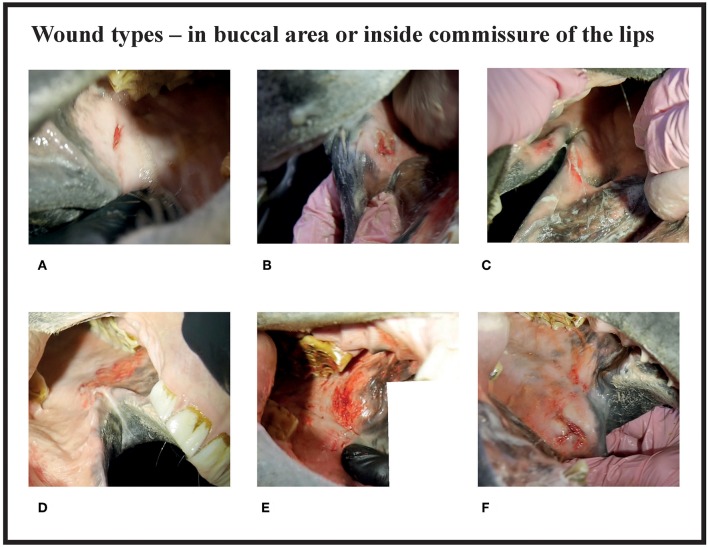
Wound types and their points in buccal area or inside commissure of the lips. **(A)** 4 points, **(B)** 6 points, **(C)** 6 points, **(D)** 8 points, **(E)** 10 points, **(F)** 10 points.

**Figure 3:**
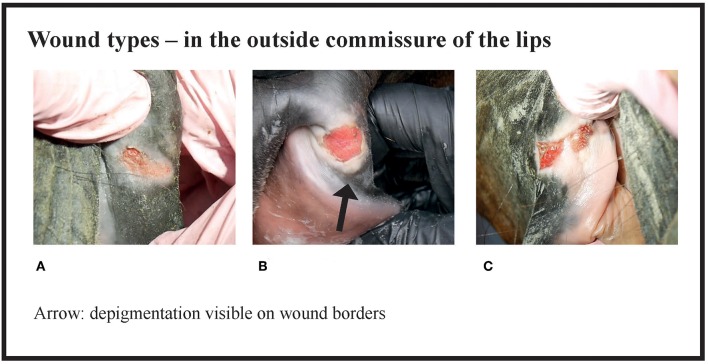
Wound types and their points in the outside commissure of the lips. **(A)** 6 points, **(B)** 6 points, **(C)** 8 points.

**Figure 4:**
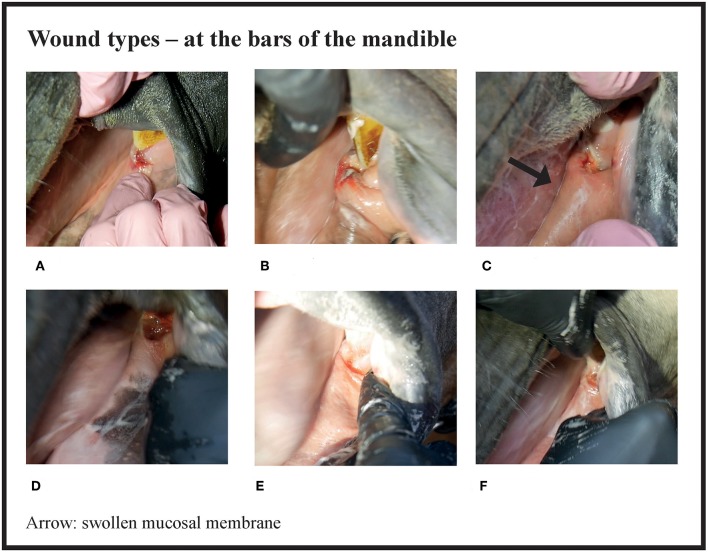
Wound types and their points at the bars of the mandible. **(A)** 4 points, **(B)** 6 points, **(C)** 8 points, **(D)** 8 points, **(E)** 8 points, **(F)** 8 points.

**Figure 5:**
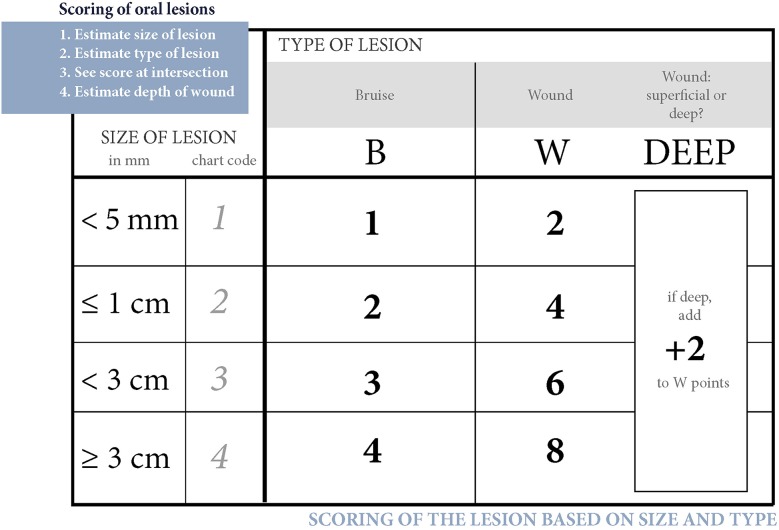
Scoring of oral lesions. Points for each acute lesion are added together to form the acute lesion score.

Since many horses had several lesions, we established a score that considered all lesions in each horse. Points were given for every acute lesion that was detected, then added up to form a total score for the horse. This acute lesion score combines the number, size, quality (bruise or wound), and depth (superficial or deep) of acute lesions. Old lesions were recorded separately. A bruise (syn. contusion, hematoma) was determined as a discoloration of a superficially intact mucosa. Bruises were given points from 1 to 4 according their size (maximum width) as follows: <0.5 cm = 1 point; 0.5−1 cm = 2 points; >1 cm but <3 cm = 3 points; 3 cm or larger = 4 points. A lesion was determined as a wound if the mucosal surface was damaged. Wounds were visually classified as deep if there was extensive damage to the submucosal tissue, or superficial if the damage was less extensive. Wounds were given points from 2 to 8 according to their size as follows: <0.5 cm = 2 points; 0.5−1 cm = 4 points; >1 cm but <3 cm = 6 points; 3 cm or larger = 8 points (Figures 1–**4**). An additional two points were added for deep wounds. Points for each lesion were added together to form the acute lesion score (**Figure 5**).

Many of the lesions were assessed as mixed-type. These lesions were graded according to the most severe lesion type present. For example, if a bruise and a wound were present in the same lesion, the lesion was graded as a wound (Figure 2C). If an old and an acute lesion were present in the same lesion, the lesion was graded as acute. If the wound borders were depigmentated (Figures 3B,C) or wound margins were thickened (Figure 2B)—both signs of an old and chronic lesion—but if the wound was red or pink and not fully healed, the lesion was graded as an acute lesion. In some cases, a wound at the bars of the mandible was accompanied by swelling (Figures 4B,C). We did not systematically record redness and swelling at the bars, but we noted both in some horses without bruises or wounds.

The horses were divided into four groups (A—D) according to their acute lesion score as follows: Group A (no acute lesions) = horses with 0 points; Group B (mild lesions) = horses with 1–2 points; Group C (moderate lesions) = horses with 3–11 points (although this excluded horses with eight points for a single lesion); Group D (severe lesions) = horses with 12 or more points (including horses with eight points for a single lesion).

### Old Lesions

**Figure 6:**
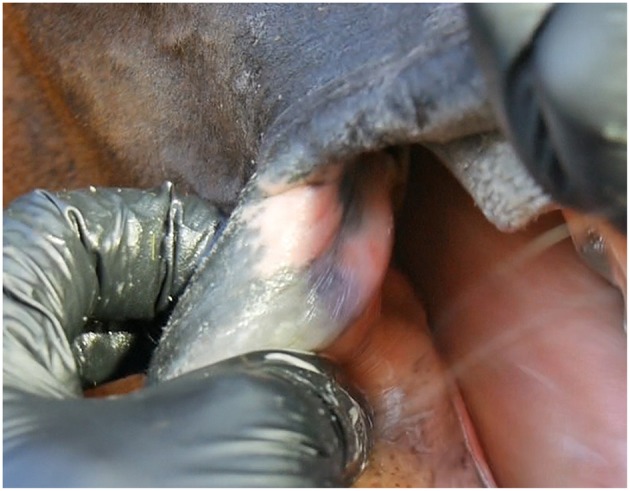
Depigmentation in the outside commissure of the lips. Plain depigmentation without scarring and thickening of the skin.

The number and type (depigmentation, old wound, old bruise or scar) of old lesions were recorded, but not included in the acute lesion score. The size of old lesions was not recorded. A bruise was evaluated as old if the red color was faded. Depigmentation (acquired leukoderma or hypopigmentation) of the outside commissures of the lips was recorded. This lack of dark pigment at the lip commissures is caused by previous inflammation or prolonged pressure from the bit (8, 9) (Figure 6).

### Bleeding

Visible blood outside the mouth, on the wound, or on the bit was recorded, but not included in the acute lesion score.

### Photographs

Early in the study, we noticed that it is difficult to take oral photographs of horses when they are out of breath. Therefore, video recordings (Panasonic DMC-GX7; lens H-FS14140, 14–140; settings: STD, MP4, 1,920 × 1,080 50 p 28 Mbps, recording mode: M (movie), exposure: P, light metering method: multiple, automatic focusing ON, optical zoom only) of the typical lesions were taken for lesion severity scale documentation purposes. From the video files, a focused frame was saved as a JPG image file using a video editing software (Adobe Premiere Pro CC 2017).

### Statistical Analysis

Statistical analyses (mean, min, max, standard deviation) were performed with Stata IC version 15 (Stata Corporation, Texas, Usa) and Microsoft Office Excel 2007.

## Results

**Figure 7:**
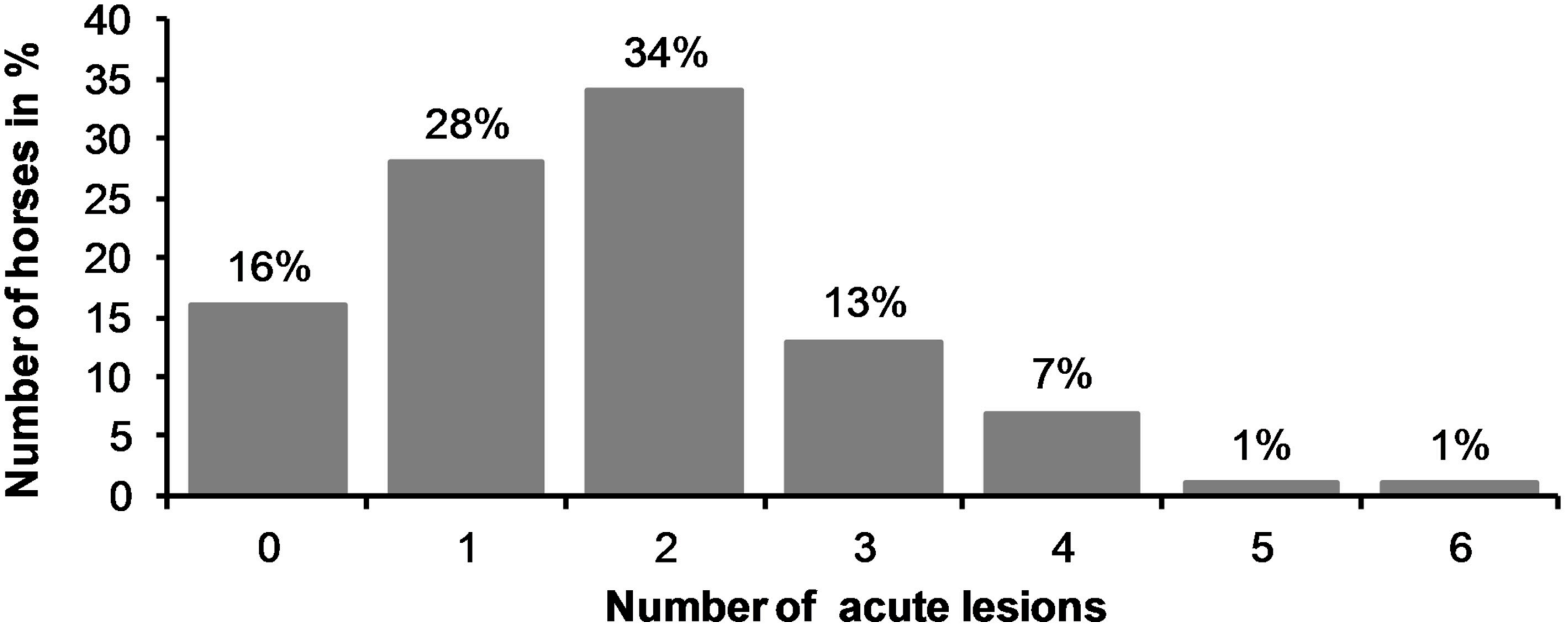
Number of acute lesions in all horses.

Only 12% (32/261) of all the horses examined had no acute or old mucosal lesions in the bit area, while 84% (219/261) had acute lesions in the bit area and 4% (10/261) had only old lesions. Among the 261 horses, 147 (56%) had more than one lesion (Figure 7). The highest number of acute lesions observed in an individual horse was six. The most common number of lesions was two. In total, 528 lesions were recorded, 452 of which were graded as acute in the bit area. During the race, single-jointed bits were the most common bit type (75%, 195/261 horses), followed by a straight bit type (18%, 48/261) and a double-jointed bit type (7%, 18/2019). The number of horses examined in this study represents 3.6% of all the trotters (*n* = 7,261) that competed in 2017 in Finland (10).

**Figure 8:**
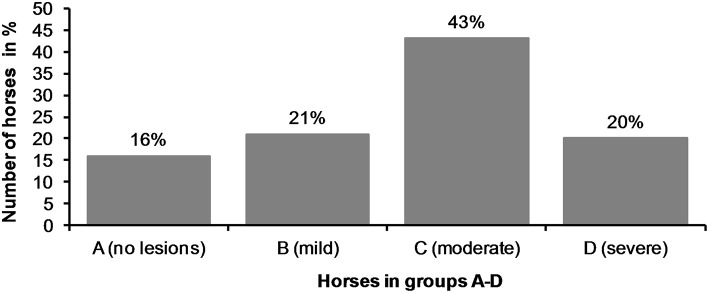
The horses (*n* = 261) were divided in four different groups (A—D) according to their acute lesion score.

**Figure 9:**
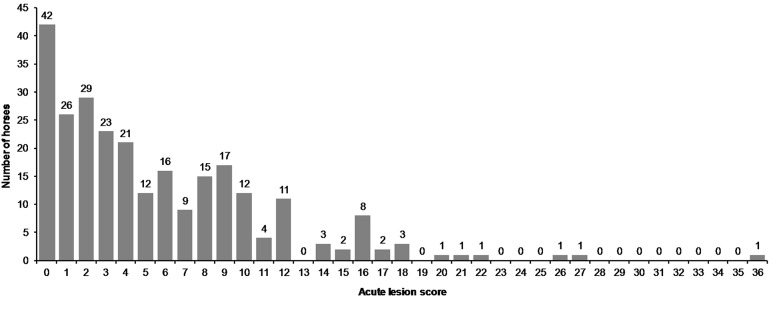
Acute lesion scores in all horses.

There were no acute lesions in 16% (42/261) of the horses (Group A). We found that 21% (55/261) of the horses had mild lesions (Group B), which meant they either had a 1 cm bruise or two bruises <0.5 cm (Figures 1A–C). 43% (113/261) had moderate lesions (Group C), and 20% (51/261) had severe lesions (Group D) (Figure 8). Group D included, for example, a horse with a single 3 cm or larger superficial wound (Figure 2D), a horse with a single deep wound larger than 1 cm (Figure 4D), and a horse with a single 1 cm bruise, a single 3 cm bruise and a single wound larger than 1 cm (Figures 1C,E, 3A). Acute lesion scores ranged from 0 to 36 (Figure 9). Among all horses, the mean acute lesion score was 5.6 (SD 5.6).

### Lesion Types

#### Bruises and Wounds

**Figure 10:**
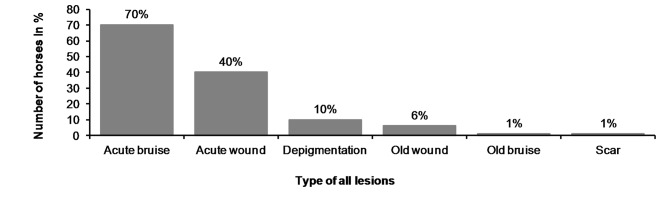
Type of the lesions in horses.

In the bit area, 70% (182/261) of the horses had bruises, and 40% (104/261) had wounds (Figure 10). The wounds had different appearances. The most common type was *abrasion*, a superficial injury to the mucosa (Figure 2D). Other types were *laceration*, a full thickness injury to the mucosa characterized by tearing of the tissue (Figure 4D), and *incision*, a clean cut wound longer than its width (Figure 2A). Deep wounds had a crater-like appearance (Figures 4B–F) or extensive damage to the submucosal tissue (Figures 2E,F).

### Old Lesions

**Figure 11:**
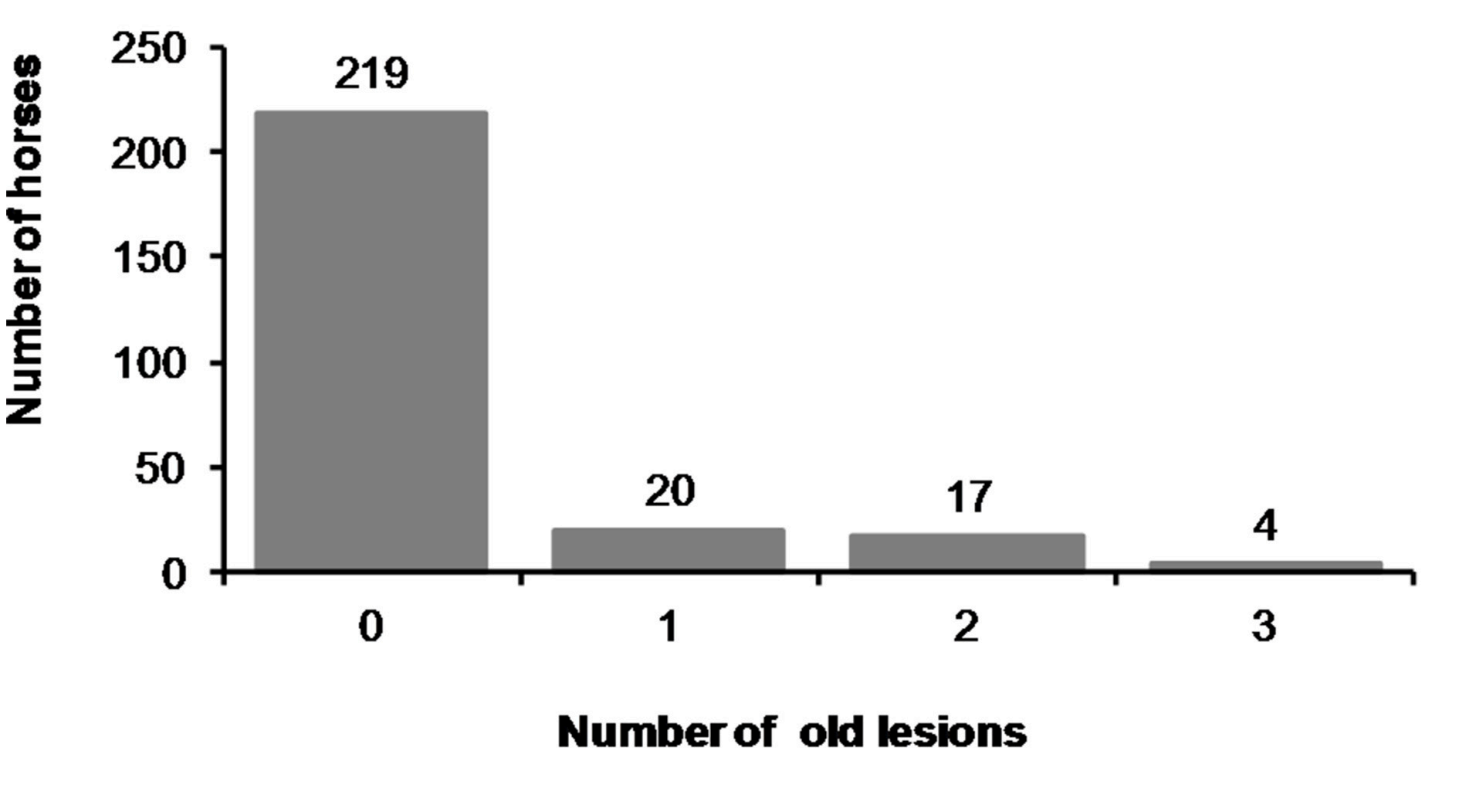
Number of old lesions.

We found old lesions in 16% (41/261) of the horses, which were characterized by depigmentation on the outside commissures of the lips, scars, old wounds, or old bruises (Figure 10). Ten horses (4%) had only old lesions and 31 horses (12%) had old lesions together with acute lesions. The number of old lesions in any single horse ranged from 0 to 3 (Figure 11).

### Location of Acute Lesions

**Figure 12:**
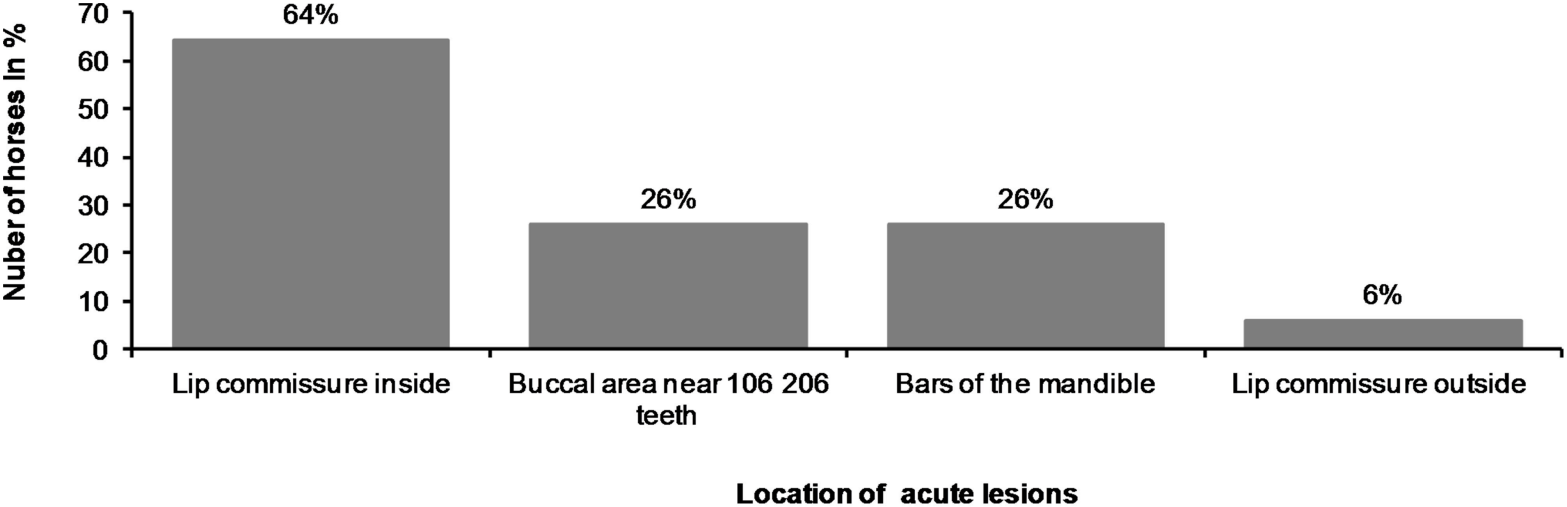
Location of acute lesions in all horses.

The most common place for a lesion was the inside commissure of the lips, where 64% of all horses had lesions. The location of lesions is presented in Figure 12. In some cases, the lesion extended from the inside commissure of the lips to the buccal area (Figures 2D–F). The location of such lesions was recorded as inside commissure of the lips.

### Tongue and Hard Palate Lesions

Nine horses had tongue lesions. Four had bitten their tongue (1.5%), three had bruises under the tongue (1.1%), and two had bruises at the sides of the tongue (0.8%). Only one horse (0.4%) had a small lesion in the hard palate. Tongue and hard palate lesions (*n* = 10) were not included in the acute lesion score in this study because these lesions were located so that they were not probably related to the bit.

### Teeth

Ten horses (3.8%) had a sharp beak in 106 or 206 teeth and in five of the horses the beak may have caused worsening of the lesion (Figure 2E). In the five other horses the lesion was not adjacent to the beak.

### Bleeding

Blood was visible outside the mouth in 2% (6/261) of the horses. One of these horses had bitten its tongue. In 5% (14/261) of the horses, blood was visible on the bit once removed from the mouth, but blood was not visible on the outside of the mouth. Blood was visible on the wound in the mouth in 5% (13/261) of the horses, but not on the bit nor outside the mouth. Two of the three horses excluded from the study had blood on the bit. For those horses with blood on the outside of the mouth, the mean acute lesion score was 15.2 (min 10, max 21, SD 4.4), and for those with blood on the bit, the mean score was 12.7 (min 6, max 26, SD 5.8). Among all the horses, the mean lesion score was 5.6 (min 0, max 36, SD 5.6). Of the 14 horses with blood on the bit, 10 had severe lesions (Group D), and four had moderate lesions (Group C). Of the six horses with blood outside the mouth, five had severe lesions (Group D) and one had moderate lesions (Group C). The horse with the moderate lesions had bitten the tip of its tongue, which caused the bleeding. The three horses that got highest points (22, 27, and 36) did not have blood anywhere.

### Timing of the Next Race Performance

In total, 20% of the horses (51/261) had severe lesions (Group D). Of those, 65% (33/51) competed again within 2 weeks, 13 competed within 1 week, and two competed again the following day.

## Discussion

We found a high occurrence of oral lesions in Finnish trotters examined. Over half of the horses (56%) had more than one acute lesion in the bit area. In fact, many had three to four lesions, and in some cases, as many as five or six. This scoring system enables an accurate evaluation of overall damage to the oral structures in the bitting area. In this respect, our method differs from previous studies on Icelandic horses, where the score was based only on the most severe lesion detected (1).

The horses were divided into four groups on the basis of their acute lesion score. Group A included horses with depigmentation of the corners of the lips, and/or an old scar or bruise, but without acute lesions considered to cause acute pain. Group B included horses with mild lesions that might potentially cause discomfort or pain, but would likely heal quickly, and therefore have minor significance for the welfare of the animal. In contrast, Group D included horses with either multiple lesions or large and deep lesions that likely cause considerable pain and heal slowly. The cut-off value for Group D was selected to include the horses with the most severe and potentially most painful damage. Group C included moderately affected horses. However, some horses with potentially painful injuries fell into Group C, such as the horse in Figure 3B. In the study of Icelandic horses, a lesion was graded as severe if it was an ulcer larger than 1 cm in diameter, with inflammation and/or soreness of the mucosa, or prominent thickening of the bars (1). In our study, some of the horses with superficial wounds larger than 1 cm fell into the moderate group (Group C), but if the horse had a deep 1 cm wound or multiple moderate lesions it fell into the severe group (Group D). Thus, in our study, both Groups C and D include horses with potential welfare problems, but the most severe cases are reflected in Group D.

Our examination method is not a substitute for a full oral or dental examination where sedation, mouth gag, and mirror are essential. Nevertheless, this quick and practical technique allows information to be acquired regarding the rostral part of the oral cavity that is potentially affected by the bit. During the examination, the horses experienced a slight and brief inconvenience while their tongue was being held. In the study by Odelros and Wattle (5), the mouth was similarly evaluated without sedation, but with a mouth gag. They flushed the mouth with tap water before the examination, which can improve visibility if the horse has a lot of mucus or food in the mouth.

In our study, 84% of the horses had acute lesions in the bit area after the race performance. This result is similar to a study of Swedish trotters, which reported lesions in 88% of horses (5). In Icelandic horses, 60% had lesions after a competition in 2012. A follow-up study reported 33% in 2014, and 43% in 2016 (1, 2). In our study, 20% of the horses had severe lesions, which is more than in Icelandic horses (8%). However, direct comparison of the results is difficult due to the different grading systems (1).

Of the trotters examined in our study, 6% had acute lesions in the outside commissures of the lips. This result is similar to the 9.2% of riding horses reported in a Danish study (6). Broodmares (*n* = 20) not using a bit did not have lesions in the lip commissures (7), indicating that this type of lesion is likely due to the use of bits. In the study by Mata et al. (4), polo ponies (*n* = 50), and racehorses (*n* = 50) had 15 and 53 commissure ulcerations, respectively. The racehorses had a significantly higher prevalence of commissure damage and a higher severity grading when compared to the polo ponies. Assuming that a horse can have a maximum of two commissure ulcers (one per side), the number of racehorses affected by this type of lesions would have been at least 25%, which is much higher than in our study. We cannot, however, directly compare our results to the Mata et al. (4) study because different grading systems were used and because the prevalence of lesion per horse was not reported and the result may contain both inside and outside commissure lesions.

We found that 26% of the trotters had lesions in the bars of the mandible. This is comparable to the 31% of Icelandic horses that had lesions after a competition (1). On the other hand, in the study by Tell et al. (7) of riding horses and broodmares, no horses had ulceration at the bars (*n* = 113). We found that it was important to examine and palpate carefully the area near the second lower premolar (306, 406), since we noticed that lesions in that area were hard to detect. We did not observe bone spurs or swellings of the bone at the bars of the mandible, but the possibility of these lesion types should be kept in mind when examining the rostral part of the oral cavity (4, 11). In the study by Mata et al. (4), polo ponies (*n* = 50) and racehorses (*n* = 50) had 28 and 30 bone spurs, respectively, in the mandible bars. No other lesions in the bars were mentioned. In future studies, lesion score points could be given for obvious swelling of the mucosa or the bone on the mandible bars.

In our study, 26% of the horses had acute lesions in the buccal area near the maxillary 06 teeth, which is more than in broodmares (5%), but less than regularly ridden horses in Sweden (56%) (7). Some of the buccal lesions and lesions extending from the inner lip commissure to the buccal area may be related to sharp enamel points of 106 and 206 teeth, if present (Figures 2A,E). A driver pulling at the reins may cause the mucous membrane to glide over these teeth with increased pressure. However, the majority of the lesions in our study were not near these potentially sharp enamel points, and therefore likely to be related to the bit rather than pressure from sharp enamel points. Doherty et al. (12) have studied noseband tightness in other equestrian sports and they found that only 7% of the horses had a noseband in the two fingers classification, which is the general recommendation. It is possible that sometimes noseband or other trotters' equipment's might press mucosal membranes against the teeth and contribute to lesions, but it has not been studied.

The oral mucosa consists of stratified squamous epithelium (mucosal epithelium) and an underlying connective tissue, called the *lamina propria* (13). Since the mouth is the gateway to the alimentary and respiratory tract, the oral mucosa is densely innervated in order to monitor all entering substances. Free nerve endings are found in the mucosal epithelium and *lamina propria*. The sensation of pain is initiated by a noxious stimulus, such as a mechanical force causing tissue damage (13, 14). It is thus likely that lesions in the oral mucosa cause pain to the horse. Pain acts as an important protective warning system to minimize tissue damage (14–16). The horse is a flight animal, and its reaction to noxious stimuli is to escape the source (17). After experiencing a painful event, the horse can try to alter its behavior by learning to avoid potentially painful stimuli (16). Many trainers were surprised to learn that their horse had severe lesions. Signs of pain in horses are not always well-recognized, even though pain affects the horses' behavior and facial expressions (18–21). When the signs are frequently witnessed, such as head tossing, mouth opening or tongue lolling, people might begin to regard such abnormal behavior as normal (20). If tissue damage is not prevented, the injured tissue causes inflammatory pain. In this state, sensitivity is increased such that stimuli that would not normally cause pain will cause it. If not treated, inflammatory pain can cause allodynia (reduced threshold to pain) or hyperalgesia (increased response to pain) (14, 15). In this study, we did not evaluate soreness or pain to palpation, since it would have been difficult to evaluate on horses with a high sympathetic tone after a race performance. Interestingly, the two horses with the highest acute lesion scores (27 and 36) were extremely difficult to examine. The horse that received 27 points appeared to have “electric shocks” when its muzzle was touched. Two of the three horses that were too difficult to examine and were excluded from the study, had blood on the bit. We suggest that these difficulties during examination and extraordinary behavior were related to oral pain. Cook and Kibler (3) compared the behavior of 66 horses with and without a bit. The study was based on a questionnaire to riders, who had switched from a bitted to a bit-free bridle. From the answers, 69 pain signals were evaluated, and they noticed a 43−100% reduction in pain signals in 65 horses when ridden without the bit. Minimizing injuries and pain by rapid diagnosis and treatment are a part of the Five Domains of animal welfare (22, 23). Even slight discomfort can cause the horse to focus on the pain rather than on performance (16, 24).

Persistence of the inflammatory response delays wound healing (13). Foreign material in the wound, such as dirt, debris, and sutures can cause an intense inflammatory reaction that interferes with normal wound healing. A bit can be considered as a “foreign material” in the mouth, potentially preventing wound healing (25). On the other hand, profuse blood supply and the moist environment in the mouth enhance wound healing, compared to skin. The time required to replace all the cells in the epithelium has been estimated to be 52−75 days in the skin, 41−57 days in the gingiva, and 25 days in the buccal mucosa (13, 25). Healing of the lesions on the skin on the external lip commissures may therefore take more time. Collagen is deposited rapidly in the wound within 5–20 days, thus increasing tissue tensile strength, although as many as 150 days may be required to regain normal tissue strength (13, 25). In our study, 33 horses with severe lesions competed again within 2 weeks, and it is thus not likely that their lesions were healed completely before the next race. We thus suggest that when lesions, especially severe ones, are recorded, there is a need for careful evaluation of when the horse can be deemed fit for competition again.

The presence of a veterinarian in harness racing events in Finland is regulated by the Animal Welfare Act (26). According to the regulations in place during the preparation of this manuscript (2019), the veterinarian may remove a horse from the race or order an oral examination and a health certificate examination before it is allowed to compete again, if it is noticed that the equipment has damaged the horse (27). However, as we have shown in this study, the absence of blood on the outside of the mouth does not rule out severe lesions inside the mouth. Moreover, it is often suggested that bleeding from the mouth is due to the horse biting its tongue. However, we found that four horses had bitten their tongue and only one horse bled from the tongue.

One explanation for the high occurrence of lesions in harness racing may be the nature of the competition. Typically, 12−16 horses run together for a distance of 1,600−2,600 m. The horses are highly aroused, and the drivers control the horses via reins and bits. Since it is not desirable for the horses to fatigue during warm-up or early in the race, drivers might hold the reins with greater tension. The horses that have been trained to respond to stronger aids may have more oral injuries than horses given lighter aids (4, 28). We did not study the amount of force applied to the reins, but it is recommended in future studies.

One limitation of our study is that the horses were not examined before the race, since we did not want to disturb the competitors. Björnsdottir et al. (1) examined 77 horses before and after the competition. Of these, 43% horses had lesions already before, and 60% after the competition. Specifically in the bar region, however, there was a clear increase (8–31%) of lesions after the competition (1). Based on the acute clinical appearance of the lesions in our study, many were likely to have been acquired during the racing event, either during the warm-up or the actual race. Alternatively, the racing event may have worsened pre-existing lesions.

## Conclusions

In conclusion, soft tissue lesions in the bit area were a common finding after a race performance in Finnish trotters examined. Lesions are easily left unnoticed, since they are inside the mouth and usually do not bleed. Importantly, while blood on the bit is a strong indication that the horse has severe lesions inside the mouth, the absence of blood on the bit and especially outside the mouth does not rule out severe injuries inside the mouth. The scoring system described here is practical, fast, and well-tolerated by the horses, and can be used to evaluate the severity of lesions at the race track. This study paves the way for future work in oral health of trotters.

## Data Availability

The datasets for this manuscript are not publicly available because the data was collected during a welfare program for trotters, conducted by The Finnish Trotting and Breeding Association (Suomen Hippos ry). Requests to access the datasets should be directed to kati.tuomola@helsinki.fi.

## Ethics Statement

The study did not include procedures to animals of a type that requires formal approval from an animal ethics committee. The study was, however, considered ethically acceptable by the University of Helsinki Viikki Campus Research Ethics Committee (Statement 8/2018). The study information was published as an announcement in the national newspaper for trainers (Hevosurheilu) and on the internet page of The Finnish Trotting and Breeding Association (www.hippos.fi) prior to the study. Anonymity of the trainers and drivers was maintained. During the examination, the horses experienced a slight inconvenience when the tongue was held, which lasted no more than 1–2 min. In general, the horses tolerated the examination well. The examination was ceased and the horse was excluded from the study if it was difficult to examine (three out of 264 horses).

## Author Contributions

KT contributed to the study design, performed the oral examinations, data collection and analysis, and preparation of the manuscript. NM-K recorded all findings, contributed to data collection and analysis, video recordings, and preparation of the manuscript. MK-W, AM, and AV contributed to interpreting the results and preparation of the manuscript. All authors read and approved the final manuscript.

### Conflict of Interest Statement

The authors declare that this study received funding from Suomen Hippos ry. The data was collected during a welfare program for trotters, conducted by Suomen Hippos ry. The funder informed the trainers of the study on their website and in their newspaper. The funder approved the proposed data collection method but had no further role in the study design, collection, analysis or interpretation of the data or preparation of the manuscript. The decision to submit the report for publication is made by the authors, and approved by the funder. KT works as a veterinarian in races at Porin Ravit Oy, which is one of the tracks, where horses were examined, but she was not on duty during the research period. The authors declare that the research was conducted in the absence of any commercial or financial relationships that could be construed as a potential conflict of interest.

## Supplementary Material

Video 1 Oral examination. An educational video on how the horse owners and trainers could examine the rostral part of the mouth for lesion prevention or early detection.

Data Sheet 1 Oral examination form. A modified version of a former Vet Form 2 from the International Federation of Icelandic Horse Associations.
