# Supplementary material for: Oral Lesions in the Bit Area in Finnish Trotters After a Race: Lesion Evaluation, Scoring, and Occurrence
Source: Front Vet Sci. 2019 Jul 12;6:206. doi: 10.3389/fvets.2019.00206 (PMC6640207; doi:10.3389/fvets.2019.00206)
Supplement: Data Sheet 1 — Oral examination form. A modified version of a former Vet Form 2 from the International Federation of Icelandic Horse Associations. [file Data_Sheet_1.PDF]

ORAL EXAMINATION FORM AFTER A RACE   No.________

Trot Track____________________________ Date__________________Breed__________

Race______/_____Horse______________________________Gender_______Age_______

Area	Lesion	Size	Depth	Bleed.	Points
Right buccal mucosa	bruise / fresh wound / old wound/ scar	1 / 2 / 3 / 4	superficial / deep	B	
Right lip corner inside	bruise / fresh wound / old wound/ scar	1 / 2 / 3 / 4	superficial / deep	B	
Right bar	bruise / fresh wound / old wound/ scar	1 / 2 / 3 / 4	superficial / deep	B	
Right lip corner outside	bruise / fresh wound / old wound/ scar/depigmentation	1 / 2 / 3 / 4	superficial / deep	B	
Left buccal mucosa	bruise / fresh wound / old wound/ scar	1 / 2 / 3 / 4	superficial / deep	B	
Left lip corner inside	bruise / fresh wound / old wound/ scar	1 / 2 / 3 / 4	superficial / deep	B	
Left bar	bruise / fresh wound / old wound/ scar/depigmentation	1 / 2 / 3 / 4	superficial / deep	B	
Left lip corner outside	bruise / fresh wound / old wound/ scar	1 / 2 / 3 / 4	superficial / deep	B	
Sides of the tongue	bruise / fresh wound / old wound/ scar	1 / 2 / 3 / 4	superficial / deep	B	
Tip of the tongue	bruise / fresh wound / old wound/ scar	1 / 2 / 3 / 4	superficial / deep	B	
Palate	bruise / fresh wound / old wound/ scar	1 / 2 / 3 / 4	superficial / deep	B	

Other observations:____

Depigmentation: lack of dark pigment at the outside corners of the mouth

Size
Size 1: less than 5mm
Size 2: max 1cm
Size 3: larger than 1 cm
Size 4: 3 cm or larger

Acute lesion score__________________
Bruise points = size of the bruise
Wound points = size x 2
Deep wound = plus 2 points

Equipments

Bit type and material____

Is the bit ”right or wrong way” in the mouth?_____________________________________

Bit thickness measured near the bitring_________________mm

Checkbit Yes No Type____

Jawstrap Yes No Material____

Overcheck Yes No Front-part_______________________back-part________________

Tonguetie Yes No Material____

Other equipment Yes No ____
